# Tonifying spleen and replenishing kidney method of traditional Chinese medicine for myasthenia gravis: A protocol for systematic review and meta-analysis

**DOI:** 10.1097/MD.0000000000025966

**Published:** 2021-05-28

**Authors:** Bibo Lu, Qing Ye, Yuting Pan, Jiachun Lu, Lu Li, Yuxuan Peng, Min He, Weiyin Chen, Xueping Yang

**Affiliations:** a Chengdu Eighth People's Hospital (Geriatric Hospital of Chengdu Medical College); b Hospital of Chengdu University of Traditional Chinese Medicine, Chengdu, China.

**Keywords:** meta-analyses, myasthenia gravis, protocols, tonifying the spleen and replenishing the kidney method for external treatment, traditional Chinese medicine

## Abstract

**Background:**

Myasthenia gravis (MG) is an autoimmune antibody-mediated disorder caused by dysfunction at the neuromuscular junction spreads. The main clinical features of this disease are fluctuating fatigue, and weakness of the skeletal muscles of the eyes and limbs. At present, the tonifying the spleen and replenishing the kidney method in traditional Chinese medicine has been widely used for MG. The present study was conducted to evaluate the efficacy and safety of the tonifying the spleen and replenishing the kidney method in traditional Chinese medicine for MG.

**Methods:**

The following 10 databases were searched from inception to March 2021: PubMed, Cochrane Library, EMBASE, Web of Science, Springer, China National Knowledge Infrastructure (CNKI), Wan fang, VIP Chinese Science and Technique Journals Database, the Chinese Bio Medical Database (CBM), and Baidu Scholar. The language was limited to the Chinese and English language. Merely randomized controlled trials (RCTs) were included. The Cochrane Collaboration risk-of-bias tool was used for the methodological quality assessment and risk of bias. The meta-analysis was assessed using the Cochrane RevMan 5.3 software.

**Results:**

In the present study, a meta-analysis was conducted, and RCTs that met the eligibility criteria were included. Furthermore, the different outcome indicators of different methods were objectively compared. The main outcome indicators included the effective rate, quantitative myasthenia gravis (QMG) scores, adverse events, and quality of life (QOL). The secondary outcome indicators included AchRAb, serum-related immune cells (such as CD3+CD4+cells and CD4+/CD8+cells), the traditional Chinese medicine syndrome score scale (TCMSSS), the serum interleukin-6 level, the level of IFN-γ and its mRNA, and the clinical score that contains the clinical absolute score (CAS) and clinical relative score (CRS).

**Conclusion:**

This study would provide credible evidence to determine whether the tonifying the spleen and replenishing the kidney method in traditional Chinese medicine is an effective treatment method for MG.

**Trial registration number:**

INPLASY202110097

## 1 Introduction

Myasthenia gravis (MG) is autoimmune chronic disorder, in which skeletal muscle weakness and fatigability account for the impairment of neuromuscular junctions (NMJ) transmission. Its global prevalence is 150 to 250 per million population, and its incidence rate has been increasing.^[1,2]^ Although the cause of MG remains complicated and unclear, a large number of studies have shown that some pathogenic antibodies cause the incidence of MG, such as acetylcholine receptor antibody (AchRAb), muscle-specific receptor tyrosine kinase (MuSK), and low-density lipoprotein receptor-related protein 4 (LRP4). The clinical manifestations of MG are blepharoptosis, diplopia, dysphagia, limbs weakness, breathing difficulties, and so on. These above symptoms are characterized by undulatory for the whole day. The diagnosis of the disease is based on the clinical symptoms, pharmacological examination, and nerve electrophysiology detection. At present, the main treatment for MG is drugs (glucocorticoids, immunosuppressants, cholinesterase inhibitors, intravenous immunoglobulins, and so on), plasma exchange, and surgery. Although these treatments have been effective in alleviating the symptoms of MG, the disease cannot be cured. Traditional Chinese medicine (TCM) and its related medical systems have been developed in China, and in some other parts of Asia, for thousands of years, in order to ensure the health of the people in these areas. A large number of studies have proven that the TCM decoction is a simple and effective approach to treat MG.

MG is described as “drooping eyelids” or “ flaccidity syndrome” in TCM literature. According to the TCM theory, the deficiency of the spleen qi leads to the weakness of the limbs. The deficiency of the kidney aggravates the deficiency of spleen, thereby worsening the symptoms of MG. Deng Tie-tao, who is an expert in TCM, considered that the main pathogenesis of MG was deficiency of the spleen and kidney, and that the treatment principle was to tonify the spleen and replenish the kidney.^[3]^ The treatment performed by Professor Qian Renyi for MG by tonifying the spleen and replenishing the kidney was effective, and this may be correlated to the decrease in the level of IFN-γ, IL-4, CD4+/CD8+, and other indicators.^[4]^ Therefore, the treatment of tonifying the kidney and replenishing the spleen has been used as the complementary medical treatment for MG. However, there has been no systematic evaluation of the safety and efficacy of tonifying the kidney and replenishing the spleen in the treatment of MG. Thus, a systematic review was conducted on the tonifying the kidney and replenishing the spleen method for MG, with focus on clinical evidence, according to the included randomized-controlled clinical trials (RCTs).

## 2 Methods and analysis

### 2.1 Protocol registration

The present study was registered in INPLASY, with registration number: INPLASY202110097. The Cochrane Collaboration Handbook for Systematic Reviews of Interventions^[5]^ and the Preferred Reporting Items for Systematic Review and Meta-Analysis Protocols (PRISMA-P)^[6]^ were used to report and guide the systematic review and meta-analysis protocol. Since there is no direct human involvement, there was no need for ethical approval.

### 2.2 Types of studies

Merely RCTs that reported the efficacy and (or) safety of tonifying the spleen and replenishing the kidney method in TCM and Western medicine were included. These articles were published in the English and Chinese language. Animal mechanism studies, case reports, self-pre- and post-control, or non-RCTs were excluded.

### 2.3 Types of participants

Patients with MG (according to any recognized diagnostic criteria, such as The Chinese Expert Consensus on The Diagnosis and Treatment of Myasthenia Gravis or the diagnosis criteria settled by the Myasthenia Gravis Foundation of America [MGFA]) were included. The included patients were approximately 18 years old. There was no restriction in sex, ethnicity, race, and disease stage. Furthermore, patients with severe cardiovascular diseases, mental illnesses, and similar disease were excluded.

### 2.4 Types of interventions

In the experiment group, the types of tonifying the spleen and replenishing the kidney methods of TCM for oral administration (such as Chinese herbal medicine and Chinese patent medicine) combined with Western medicine were used for the treatment of MG.

In the control group, one or more Western medicine (such as acetylcholine preparation, hormone, immunosuppressant, intravenous immunoglobulin, etc) or placebo control were used, or no therapy was performed. There were no limitations on the intervention approach.

### 2.5 Types of outcomes

The primary outcomes are listed below:

a. Effective rate;
b. The quantitative myasthenia gravis (QMG) score;
c. Adverse events;
d. Quality of life.

The secondary outcomes were, as follows:

a. The concentration of acetylcholine receptor antibody (AchRAb) in serum;
b. The change in related immune cells in the serum traditional Chinese medicine syndrome score scale (TCMSSS);
c. The serum interleukin-6 level;
d. The level of IFN-γ and its mRNA;
e. The clinical score calculated based on The Chinese Expert Consensus on The Diagnosis and Treatment of Myasthenia Gravis (the clinical score contains the clinical absolute score and clinical relative score).

### 2.6 Data sources and search strategies

A systematic literature search of articles published up to February 28, 2021 was conducted with the assistance of an experienced librarian in the following electronic databases: PubMed, Cochrane Library, EMBASE, Web of Science, Springer, China National Knowledge Infrastructure (CNKI), Wan-fang database, VIP Chinese Science and Technique Journals Database, the Chinese Bio Medical Database (CBM), and Baidu Scholar. All RCTs that used the tonifying the spleen and replenishing the kidney method in TCM, in combination with Western medicine, for the treatment of MG were collected. Merely the English and Chinese language was applied for the present study. Unpublished studies were not be sought (Table 1).

**Table 1:** Search strategy in Medline.

#1	exp myasthenia gravis/
#2	myastheni$.tw.
#3	exp Weizheng/
#4	1 or 2 or 3
#5	exp tonifying spleen and replenishing kidney /tonifying spleen kidney/ spleen/ kindey/
#6	exp traditional Chinese medicine/ Chinese Traditional Medicine/ or Medicine, Chinese Traditional/ or Drugs, Chinese Herbal/ or Plants Medicinal/ or Medicine, Herbal/ or Medicine, Oriental Tradition/
#7	(Chinese medicine$ or TCM or Traditional Medicine$ or Herb$ or Chinese Drug$ or Chinese Materia Medica$ or Plant^∗^ or Phytotherap^∗^).tw.
#8	5 or 6 or 7
#9	4 and 8
#10	randomized controlled trial.pt.
#11	controlled clinical trial.pt.
#12	randomized. ab.
#13	placebo. ab.
#14	drug therapy. fs.
#15	randomly. ab.
#16	trial. ab.
#17	groups. ab.
#18	10 or 11 or 12 or 13 or 14 or 15 or 16 or 17
#19	9 and 18
#20	exp animals/ not humans. sh.
#21	19 not 20
#22	Remove duplicates from 21

### 2.7 Data extraction

#### 2.7.1 Selection of studies

Two reviewers independently and efficiently browsed the titles and abstracts from the selected articles. The reasons for the elimination of articles were noted, and a table of eliminated articles was prepared. Any disagreements were solved by discussion with a third reviewer. The data obtained from the efficient selection of articles were, as follows:

(1) Essential information: first author, year of publication, and country;
(2) Study design: setting, inclusion and exclusion criteria, randomization method, blinding, sample size, and drop outs;
(3) Participants: sex, age, and disease duration;
(4) Methodological characteristics: title study design, MG severity, and diagnostic criteria;
(5) Details of intervention: type of intervention, the names of the traditional Chinese prescriptions for treatment, duration of treatment, and follow-up time;
(6) Outcome measures.

#### 2.7.2 Dealing with missing data

For missing or insufficient data, the authors were contacted to request for adequate data by email or telephone. If the missing data could not be obtained, the trails with missing data were detached from the data synthesis. Furthermore, a sensitivity analysis was performed to determine the impact of the missing data.

### 2.8 Risk of bias assessment and quality of selected studies

The methodological quality of each included study was assessed by 2 reviewers using the Cochrane Collaboration's risk of bias tool. Attention was given on the following terms: generation of random sequence, allocation concealment, blinding of participants and personnel, incomplete outcome data, duration of follow-up, selective reporting, and other bias. These terms were be categorized into 3 levels: low risk, unclear risk, or high risk. Any disagreements between the reviewers were settled by discussion, or seeking advice from a third reviewer.

### 2.9 Statistical analysis

If a meta-analysis was possible, the data synthesis was presented using the RevMan V.5.3 statistical software. The statistically data were meaningful when *P* < .05. The SMD with 95% confidence interval (CI) was used to evaluate the continuous outcomes, and the RR with 95% CI was used to evaluate the dichotomous data. The fixed effects model (*I*^*2*^ < 50%) was used to estimate the RR and MD. The random effects model (*I*^2^ > 50%) was considered for the indication of substantial statistical heterogeneity, and used for the synthesis of the data, and the subgroup analysis or sensitivity analysis. Funnel plots were drawn for obvious publication bias.

### 2.10 Sensitivity analysis

The sensitivity analysis was conducted to evaluate the robustness and reliability of the pooled results. If adequate data were available for analysis, a sensitivity analysis for the primary outcomes was conducted to test the strength of the review conclusions, which included the quality of the methods and studies, and the impact of the sample size and missing data.

### 2.11 Publication bias

If >10 studies were included, the funnel plot would be used to determine the publication bias. Egger test and Begg test were used to quantitatively assess the publication bias using the Stata V.16.0 software. The results were estimated based on the Cochrane Handbook for Systematic Reviews of Interventions.

### 2.12 Confidence in cumulative evidence

The strength of evidence was evaluated based on the Grading of Recommendations assessment, Development, and Evaluation system. The evidence was classified into 4 levels: high, moderate, low, or very low.

### 2.13 Selection of studies

**Figure 1:**
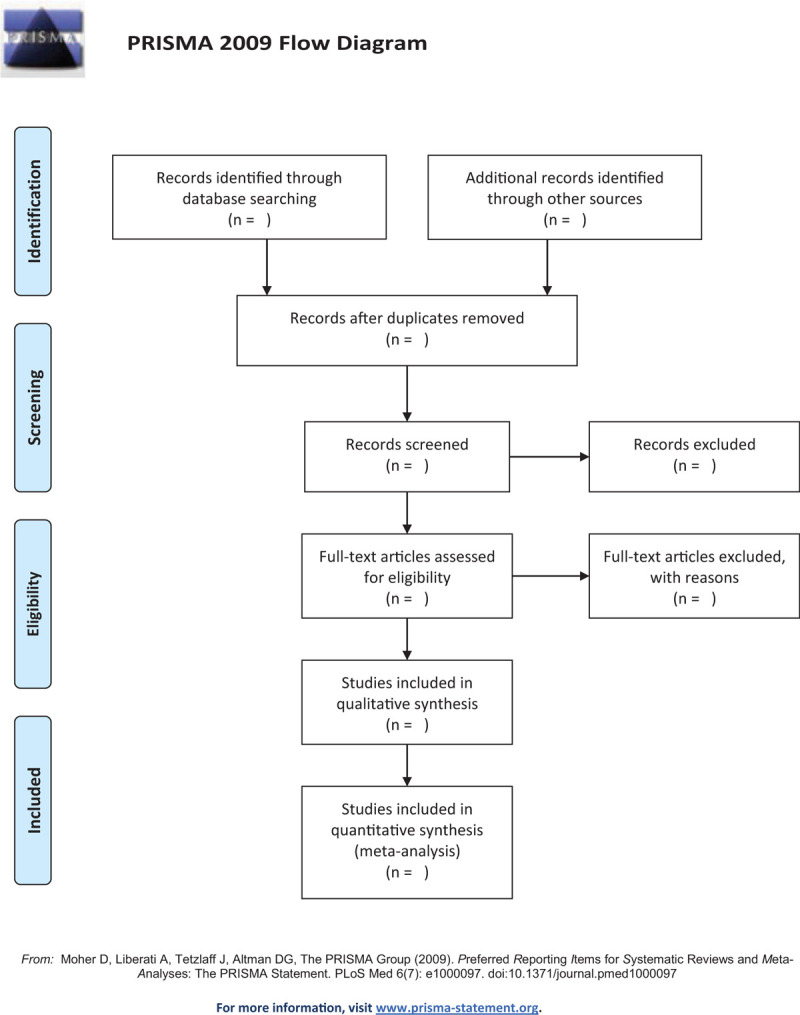
Flow diagram of study selection process.

Two review authors, who have been trained in systematic review techniques, independently searched and assessed the titles and abstracts of the potential studies identified through the search strategy for eligibility. If the eligibility of a study was inexplicit from the title and abstract, the whole article was browsed. Studies that did not meet the inclusion criteria for the present review were excluded, and the reasons for the exclusion of these articles were recorded. If there was any disagreement, this was resolved through the consensus of all authors. The Preferred Reporting Items for Systematic Reviews and Meta-Analyses (PRISMA) study flowchart was used to record the screening process (Fig. 1).

## 3 Discussion

MG is a chronic disease that seriously affects the quality of patients with MG. Western medicines, such as acetylcholine preparation, hormone, immunosuppressants, and intravenous immunoglobulin^[7]^ have been used to suppress immune system disorders and control the symptoms. In particular, new molecules with selective action in targeting compounds of the immunological system have been identified. However, it remains difficult to cure MG, and some adverse reactions continue to appear.^[8]^ Hence, more researchers on TCM for MG have been published in recent years. In particular, the tonifying the spleen and replenishing the kidney method in TCM for MG has been applied for patients with spleen and kidney deficiency. According to the TCM theory, the spleen is responsible for the transportation and transformation of Qi and blood, and the distribution of water and grain. The deficiency of the spleen qi prevents the transportation and distribution of water and grain, which are subtle to the muscles and veins of the limbs, and the loss of water and grain subtle support leads to weakness of the limbs. The kidney is the place of yuan Yin and yuan Yang. The function of the spleen to transport water and grain depends on the warmness of the yuan-yang in the kidney, and the essence of the kidney depends on the filling and nourishing of the water and grain from the spleen. Therefore, the treatment of tonifying the spleen and replenishing the kidney has been used as a complementary medical treatment for MG. However, no systematic reviews and meta-analyses on the tonifying the spleen and replenishing the kidney method for MG have been published, to date. The evaluation of the effectiveness and safety of the tonifying the spleen and replenishing the kidney method in TCM for MG would offer better evidence for clinical treatment.

## Author contributions

**Conceptualization:** Xueping Yang, Min He.

**Data curation:** Lu Li, Yuxuan Peng.

**Formal analysis:** Lu Li, Yuxuan Peng.

**Funding acquisition:** Weiyin Chen.

**Investigation:** Qing Ye, Yuting Pan.

**Methodology:** Bibo Lu, Jiachun Lu.

**Project administration:** Bibo Lu, Jiachun Lu.

**Resources:** Xueping Yang, Min He.

**Software:** Yuxuan Peng, Qing Ye, Yuting Pan.

**Supervision:** Qing Ye, Yuting Pan.

**Validation:** Weiyin Chen.

**Visualization:** Xueping Yang, Min He.

**Writing – original draft:** Yuxuan Peng, Bibo Lu, Jiachun Lu.

**Writing – review & editing:** Qing Ye, Yuting Pan.
